# Genome Sequence of the *Gordonia rubripertincta* Phage Yucky (Cluster CT)

**DOI:** 10.17912/micropub.biology.001981

**Published:** 2026-04-16

**Authors:** Caitlin Carter, Paxton R. Evitts, Cheyenne Helton, Madeline F. Kaiser, Jehee F. Lee, Mackenzie M. Peters, Cade Riley, Mackenzie L. Sanders, Chad Walker, Danielle K. Watt, Pamela L. Connerly, Elizabeth E. Rueschhoff

**Affiliations:** 1 School of Natural Sciences, Indiana University Southeast, New Albany, IN, US; 2 School of Social Sciences, Indiana University Southeast, New Albany, IN, US

## Abstract

Novel bacteriophage Yucky infects *Gordonia rubripertincta* , a bacterium known for its bioremediation potential. Phage particles have a siphovirus morphology and form 1.2 mm ± 0.5 mm-wide plaques (n=10). The genome of Yucky is 47,803 bp in length and encodes 74 protein-coding genes. The phage was assigned to cluster CT based on gene content similarity of at least 35% to actinobacteriophages. Thirty-nine gene products have putative functions, including genes involved in lysis, such as two lysin A domain genes as well as a lysin B gene.

**Figure 1. Plaque and virion morphology.** A. Plaque Morphology of Gordonia Phage Yucky. The plaque size of Yucky is variable, but average approximately 1.2 mm ± 0.5 mm in size (n=10). Shown in a segment of a standard 100 mm plate. B. Transmission Electron Micrograph of Yucky. Negative staining with uranyl acetate (2%) reveals phage particles with a capsid diameter of 63.7 nm ± 2.6 nm and a tail length of 270.6 nm ± 17.8 nm (n=5). Measurements of plaque diameter, capsids, and tails were made utilizing ImageJ software (v1.54k) (Schneider et al. 2012).

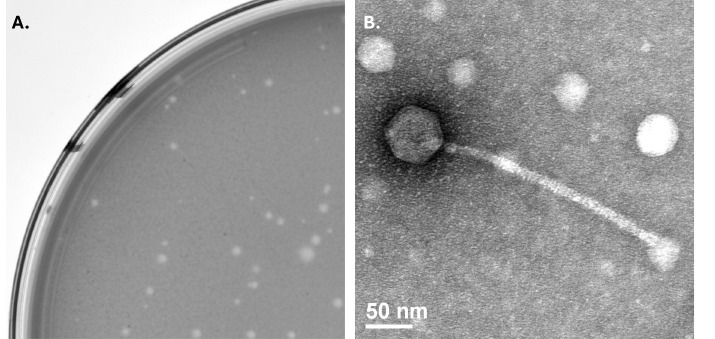



## Description

Strains of *Gordonia rubripertincta* have been characterized as bioremediators of various environmental pollutants. For example, *G. rubripertincta* strain 112 degrades aromatic and aliphatic compounds (Frantsuzova et al. 2023), while *G. rubripertincta* CWB2 degrades styrene (Lienkamp et al. 2021). Identifying and studying bacteriophages that infect these bioremediators may be informative in controlling and utilizing these bacteria in the environment.

Bacteriophage Yucky was isolated from soil collected in Jeffersonville, IN, USA (Global Positioning Coordinates [GPS]: 38.35299 N, 85.71948 W). A mixture of soil and PYCa media was incubated for approximately 2 hours at 26 °C and shaken at approximately 250 RPM. The mixture was then centrifuged, and the supernatant was filtered using a 0.2-μm filter. The filtrate was inoculated with *G. rubripertincta* NRRL B-16540 to enrich for phages capable of infecting this host and incubated for approximately 5 days at 220 rpm at 26 degrees Celsius. The sample was again filtered (0.2 μm pore size) and plated using soft agar overlay. Plates were incubated for approximately 48 hours at 26 °C. Plaques formed were round and clear, measuring 1.2 mm ± 0.5 mm in diameter (n=10). Yucky was further purified through two rounds of plating before high titer lysates were collected (Zorawik et al. 2024). Negative stain electron microscopy (2% uranyl acetate) revealed phage particles with a siphovirus morphology, with a capsid size of 63.7 nm ± 2.6 nm and a tail length of 270.6 nm ±17.8 nm (n=5).

DNA was isolated from a high titer lysate of Yucky using a QIAGEN DNeasy Blood and Tissue Kit (Jakociune and Moodley 2018). The DNA was prepared for sequencing using a New England Biolabs Ultra II FS Library Kit and sequenced with an Illumina NextSeq 1000 sequencer (X-LEAP PI Kit). Raw 100 base reads were trimmed with cutadapt 4.7 (using the option: –nextseq-trim 30) and filtered with skewer 0.2.2 (using the options: -q 20 -Q 30 -n -l 50) prior to assembly (Martin 2011; Jiang et al. 2014; Wick et al. 2017; Gordon et al. 1998). Newbler v2.9 (Miller et al. 2010) and Consed v29 (Gordon and Green 2013) were used to assemble the trimmed reads and check them for completeness (Russell 2018), with an assembly coverage of approximately 3577x. The genome is 47,803-bp in length, has a GC content of 60.5%, and has genome ends characterized by 3' single-stranded overhangs 10 bases in length (5'-CGGTAGGCTT-3').

The genome of Yucky was auto-annotated utilizing Glimmer v3.02b and Genemark v2.5p (Delcher et al. 2007; Besemer and Borodovsky 2005). Manual annotation was performed using the DNA Master program v5.23.6 build 2705 (Pope and Jacobs Sera 2018; http:// cobamide2.bio.pitt.edu), BLASTp v2.16.0 utilizing the NCBI nonredundant and Actinobacteriophage databases (Altschul et al. 1990), Starterator v594 (http://phages.wustl.edu/starterator/), Phamerator utilizing Actino_Draft v594 (Cresawn et al. 2011), HHPred utilizing the PDB_mmCIF70_30_Mar, Pfam-A v37, NCBI Conserved Domains databases (CD) v3.19, and UniProt-SwissProt-viral70_3_Nov2021 databases (Söding et al. 2005), Aragorn v1.2.41 (Laslett and Canback 2004), and deepTMHMM v1.0.42 (Krogh et al. 2001). Default settings were used for all software.

Seventy-four total protein-coding genes were predicted, and no tRNA genes were identified. Of these 74 genes, 39 were assigned putative functions and 35 were hypothetical proteins. Based on gene content similarity of at least 35% to phages in the Actinobacteriophage database, Yucky was assigned to cluster CT (Russell and Hatfull 2017; Pope et al 2017). As a member of cluster CT, Yucky contains genes assigned to a majority of common functions found in all cluster CT phages (Mitchell et al. 2024) including viral structure and assembly, DNA replication and recombination, and cell lysis. The domains of the endolysin are split into two genes: one encoding the L-Ala-D-Glu peptidase domain (gene 20) and the other encoding the glycosyl hydrolase domain (gene 21). In addition, a lysin B gene was identified (gene *49* ). Yucky also contains genes less commonly found within cluster CT phages including a predicted PAPS reductase-like domain (gene *2* , found in only 4 cluster CT phages), and a predicted SprT-like protease (gene *37* , found in 18 cluster CT phages).

To date, Yucky is one of 77 sequenced phages in cluster CT. Within the cluster, Yucky is most similar to phages Vine and PotPie, with a gene content similarity (GCS) of 93.2% and 93.8%, respectively (Russell and Hatfull 2017). Relative to one another, each phage encodes short segments of unique genes; Vine genes *1* , *3* , *4* , *35* , and *46* , PotPie genes *29* , *30* , and *33* , and Yucky genes *34, 35,* and *46* are all unique. No putative genes with immunity repressor, integrase or DNA partitioning functions were identified, suggesting that Yucky is unlikely to develop lysogeny.

Nucleotide sequence accession numbers for Yucky is available at GenBank with Accession No. PV876943 and Sequence Read Archive (SRA) No. SRX28484036 .
